# Extracellular matrix complexity in biomarker studies: a novel assay detecting total serum tenascin-C reveals different distribution to isoform-specific assays

**DOI:** 10.3389/fimmu.2023.1275361

**Published:** 2023-11-22

**Authors:** James Ozanne, Mel Lewis, Anja Schwenzer, Dominic Kurian, Jeff Brady, David Pritchard, Gerry McLachlan, Colin Farquharson, Kim S. Midwood

**Affiliations:** ^1^ The Roslin Institute, Royal (Dick) School of Veterinary Studies, University of Edinburgh, Edinburgh, United Kingdom; ^2^ R&D Department Axis-Shield Diagnostics, Axis-Shield Diagnostics Ltd, Dundee, United Kingdom; ^3^ Nuffield Department of Orthopaedics, Rheumatology and Musculoskeletal Sciences, Kennedy Institute of Rheumatology, Oxford University, Oxford, United Kingdom

**Keywords:** extracellular matrix, tenascin-C, alternative splicing, citrullination, biomarker, serum, inflammatory disease

## Abstract

Serum biomarkers are the gold standard in non-invasive disease diagnosis and have tremendous potential as prognostic and theranostic tools for patient stratification. Circulating levels of extracellular matrix molecules are gaining traction as an easily accessible means to assess tissue pathology. However, matrix molecules are large, multimodular proteins that are subject to a vast array of post-transcriptional and post-translational modifications. These modifications often occur in a tissue- and/or disease-specific manner, generating hundreds of different variants, each with distinct biological roles. Whilst this complexity can offer unique insight into disease processes, it also has the potential to confound biomarker studies. Tenascin-C is a pro-inflammatory matrix protein expressed at low levels in most healthy tissues but elevated in, and associated with the pathogenesis of, a wide range of autoimmune diseases, fibrosis, and cancer. Analysis of circulating tenascin-C has been widely explored as a disease biomarker. Hundreds of different tenascin-C isoforms can be generated by alternative splicing, and this protein is also modified by glycosylation and citrullination. Current enzyme-linked immunosorbent assays (ELISA) are used to measure serum tenascin-C using antibodies, recognising sites within domains that are alternatively spliced. These studies, therefore, report only levels of specific isoforms that contain these domains, and studies on the detection of total tenascin-C are lacking. As such, circulating tenascin-C levels may be underestimated and/or biologically relevant isoforms overlooked. We developed a highly specific and sensitive ELISA measuring total tenascin-C down to 0.78ng/ml, using antibodies that recognise sites in constitutively expressed domains. In cohorts of people with different inflammatory and musculoskeletal diseases, levels of splice-specific tenascin-C variants were lower than and distributed differently from total tenascin-C. Neither total nor splice-specific tenascin-C levels correlated with the presence of autoantibodies to citrullinated tenascin-C in rheumatoid arthritis (RA) patients. Elevated tenascin-C was not restricted to any one disease and levels were heterogeneous amongst patients with the same disease. These data confirm that its upregulation is not disease-specific, instead suggest that different molecular endotypes or disease stages exist in which pathology is associated with, or independent of, tenascin-C. This immunoassay provides a novel tool for the detection of total tenascin-C that is critical for further biomarker studies. Differences between the distribution of tenascin-C variants and total tenascin-C have implications for the interpretation of studies using isoform-targeted assays. These data highlight the importance of assay design for the detection of multimodular matrix molecules and reveal that there is still much to learn about the intriguingly complex biological roles of distinct matrix proteoforms.

## 1 Introduction

Tissues are made up of cells surrounded and supported by a 3D network of secreted molecules, known as the extracellular matrix. Each tissue comprises a unique combination of matrix proteins that define its physical properties and provide positional cues to resident cells, but which is also surprisingly dynamic. An altered matrix structure and content not only accompany tissue development and ageing but are also amongst the first responses during disease onset. Accordingly, changes in the matrix, measured both within tissues and in the circulation, are emerging as reliable biomarkers of disease onset, progression, and treatment response. For example, circulating levels of collagen type I fragments correlate with bone destruction in people with RA [reviewed in (1)], whilst cleavage products of collagen types III, IV, and VI in the serum accurately reflect improvement following treatment of inflammatory bowel disease (IBD) (2). However, the identification of biomarkers that effectively complement and improve the management of inflammatory disease has been slow in general. Whilst there is significant and largely untapped potential in the utility of extracellular matrix as disease biomarkers, it is important to carefully consider assay design for these molecules.

For example, tenascin-C is a pro-inflammatory extracellular matrix protein that exhibits restricted expression in healthy adult tissues but has long been known to be significantly and persistently upregulated in autoimmune, fibrotic, and metabolic diseases, as well as in cancer (3–5). Elevated levels are found both in inflamed tissues and in the circulation of patients with inflammatory diseases (**Table 1**), prompting intense interest in the development of serum tenascin-C as a clinically useful biomarker. Moreover, this molecule is a key driving factor of pathological inflammation. It is sufficient to trigger inflammation upon exogenous administration, and its deletion protects from chronic inflammation in experimental diseases including RA, IBD, fibrosis, and cancer. In addition, therapeutic antibodies targeting its pro-inflammatory activity effectively ameliorate disease in pre-clinical validation (1, 25). This intrinsic involvement in the disease process suggests the potential to use serum tenascin-C both as a mechanistic biomarker and a companion diagnostic to identify people most likely to benefit from treatment with tenascin-C-directed therapies. However, current methods for the detection of this molecule in serum samples are limited.

**Table 1:** Summary table of published data on tenascin-C as a biomarker of inflammatory disease.

Disease	Methodology employed	Key findings	Reference
AAV	ELISA of serum tenascin-C (FN III-C splice specific)	Tenascin-C was higher in active disease compared to healthy controls and patients in remission and correlated with disease activity and occurrence of lung infiltration	(Ishizaki et al., 2017) (6)
Asthma	ELISA of serum tenascin-C (FN III-C splice specific)	Tenascin-C was higher in severe asthma compared to mild asthma and was associated with clinical features such as more severe airflow limitation	(Yasuda et al., 2018) (7)
Ankylosing spondylitis	ELISA of serum tenascin-C (FN III- splice specific)	Tenascin-C was elevated in patients compared to healthy controls and correlated with markers of disease activity. Levels also correlated with response to treatment	(Gupta et al., 2018) (8)
IBD	ELISA of serum tenascin-C	Tenascin-C was higher in IBD patients compared to healthy controls and correlated with measures of disease activity. A treatment responsive decrease was also observed	(Riedl et al., 2001) (9)
	IHC for tenascin-C in colon biopsies	Thickened subepithelial tenascin-C staining shown to be an accurate marker for diagnosis of collagenous colitis	(Muller et al., 2001) (10)
IIM	IHC of tenascin-C in muscle biopsies	Tenascin-C staining pattern was shown to distinguish dermatomyositis from polymyositis	(Muller- Felber et al., 1998)
IgA nephropathy	IHC and ISPCR for tenascin-C in kidney biopsies	Tenascin-C protein staining was associated with disease chronicity while mRNA expression was associated with disease activity	(Masaki et al., 1998) (11)
JIA	ELISA of serum tenascin-C (FN III-C splice specific)	Tenascin-C was elevated in active disease compared to inactive disease and healthy controls Levels correlated with markers of disease activity and decreased with therapy.	(Shukla et al., 2015)
Kawasaki disease	ELISA of serum tenascin-C (FN III-C splice specific)	Tenascin-C found elevated in the serum compared to healthy controls and recovering patients. Levels correlated with disease parameters and poor response to intravenous immunoglobulin	(Okuma et al., 2016) (12)
Myocarditis	IHC for tenascin-C in myocardial biopsies	Tenascin-C staining was elevated in cases with active myocarditis and discriminated from patients with non-inflammatory dilated cardiomyopathy. Staining correlated with severity of histological lesions	(Morimoto et al., 2005, Tsukada et al., 2009) (13, 14)
Psoriasis	IHC of skin biopsies	Tenascin-C was shown elevated in psoriatic skin lesions although it did not correlate with disease activity	(Latijnhouw ers et al., 1998a) (15)
RA	ELISA of serum tenascin-C (FN III-C splice specific)	Tenascin-C was elevated in patients compared to healthy controls and further increased with longer disease duration. Levels also correlated with joint erosion severity and decreased in response to therapy. Baseline tenascin-C was predictive of treatment responsiveness.	(Page et al., 2012) (16)
	ELISA of serum autoantibodies targeting citrullinated tenascin-C	Autoantibodies against citrullinated tenascin-C (cTNC) had high diagnostic specificity for RA and were found in -50% of patients. cTNC autoantibodies were found to pre-date disease development and in undifferentiated disease were associated with the development of RA. cTNC autoantibodies also associated with a subset of patients with periodontitis.	(Schwenzer et al., 2015, Raza et al., 2016, Schwenzer et al., 2017) (17–19)
Scleroderma	ELISA of serum tenascin-C (FN III-C splice specific)	Tenascin-C was higher in scleroderma patients compared to healthy controls Patients with pulmonary fibrosis involvement had the highest levels.	(Brissett et al., 2012) (20)
SLE	ELISA of serum tenascin-C (FN III-B splice specific)	Serum tenascin-C was higher in patients with active disease compared to those with inactive disease and healthy controls Elevated levels predicted need to escalate therapy in response to disease flare.	(Zavada et al., 2015) (21)

References and detail on the methodology employed to measure tenascin-C in the study are also provided. Disease and assay abbreviations are detailed at the end of the table.

AS, ankylosing spondylitis; SLE, systemic lupus erythematosus; RA, rheumatoid arthritis; IIM, idiopathic inflammatory myositis; AAV, antineutrophil cytoplasmic antibody (ANCA)-associated vasculitis; JIA, juvenile idiopathic arthritis; IHC, immunohistochemistry; ISPCR, *in situ* polymerase chain reaction; ELISA, Enzyme- linked immunosorbent assay; SPECT, single-photon emission computed tomography.

References (6–14, 16, 18–24).

**Figure 1:**
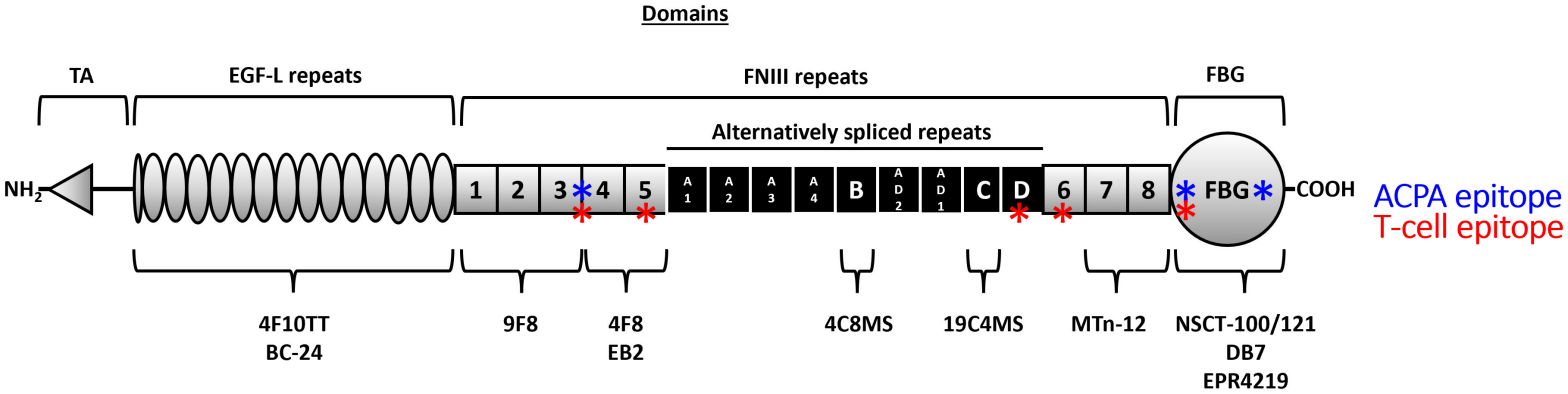
The domain structure of the human tenascin-C monomer and location of key antibody sites. Tenascin-C comprises an assembly (TA) domain, epidermal growth factor-like (EGF-L) repeats, up to 17 fibronectin III-like (FnIII) repeats, and a fibrinogen-like globe (FBG). Eight of the FnIII repeats are constitutively expressed (1–8) (grey) whilst nine of its FnIII repeats can be alternatively spliced (A1-A4, B, C, D, AD1, and AD2) (black). The location of the binding sites of antibodies used in this study is highlighted underneath the monomer, and known T (red) and B (blue) cell epitopes are denoted along the monomer length (17, 26).

Tenascin-C has a multi-modular structure comprising an assembly (TA) domain, epidermal growth factor-like (EGF-L) repeats, up to 17 fibronectin III-like (FnIII) repeats, and a fibrinogen-like globe (FBG). Tenascin-C exhibits significant isoform diversity arising from alternative splicing within nine of its FnIII repeats (A1-A4, B, C, D, AD1, and AD2) (**Figure 1**). Each of these repeats is encoded by a separate exon enabling up to 511 different potential combinations. Moreover, the protein is subject to post-translational modification by glycosylation and citrullination [reviewed in (27)]. Whilst our understanding of the biological roles of distinct tenascin-C isoforms and proteoforms is still in its infancy, it is clear that different variants engender strikingly different cellular responses. For example, the pro-inflammatory action of this protein is regulated by splicing in or out domains AD1 and AD2 which control activation of innate immune sensor toll-like receptor 4 (28). Moreover, a cryptic α5β1 integrin binding site in domain A2 activates pro-survival Akt/Bcl-2 pathways and enhances PDGF-dependent cell proliferation (29), whilst binding to α7β1 integrin via domain D promotes neurite extension (30, 31). In addition, glycosylation confers the ability of tenascin-C to bind to viral envelope proteins during HIV infection, neutralising target cell infection (32–34). The complexity of post-transcriptional/translational events that define the expressed proteoform of tenascin-C also makes the study of the tissue distribution of this molecule interesting. Examining splicing products using PCR or antibodies that recognise distinct FnIII domains reveals, for example, the enrichment of large isoforms containing all alternatively spliced domains over smaller isoforms lacking domains A1-D during tissue remodelling in embryogenesis (27). These studies also demonstrate that activated immune cells or fibroblasts from people with RA overexpress variants containing repeats BAD2AD1CD compared to contiguous BCD-containing variants found in quiescent cells or fibroblasts from healthy controls (28), whilst domain A1 is overexpressed in malignant metastatic melanoma (35).

The majority of studies measuring serum tenascin-C employed ELISAs which detect specific isoforms of tenascin-C by virtue of using antibodies that recognise either the FnIIIB or the FnIIIC domain (**Table 1** and **Figure 1**). There are currently no assays with which to accurately assess circulating levels of ‘total’ tenascin-C encompassing all splice variants. To address this, we developed a novel ELISA and immunoprecipitation method to probe total tenascin-C levels in human serum. We used these tools to assess the distribution of total tenascin-C and to determine how the total tenascin-C pool relates to levels of specific variants. To do this, we used sera from healthy controls and a cohort of people with a range of inflammatory and musculoskeletal diseases. We also screened a subset of samples with splice-specific ELISAs, as well as for autoantibody positivity, including those recognising citrullinated tenascin-C. We demonstrated the development of an ELISA that is sensitive and specific for purified tenascin-C, as well as that detected in human clinical samples. Comparison to splice-specific assays revealed weak correlations between total tenascin-C and distinct isoforms, whilst the amount of tenascin-C in RA patients’ sera did not correlate with the presence of autoantibodies against citrullinated tenascin-C. Our work showed that levels of tenascin-C variants cannot be used as a proxy for total tenascin-C, and this new assay provides the opportunity to better examine the distribution of this molecule and known variants in health and disease.

## 2 Materials and methods

### 2.1 Materials

NSCT-121 antibody was provided as a kind gift by Nascient Ltd, Cambridge, UK, and was generated as previously described (36). All other tenascin-C antibodies were sourced as detailed in **Table 2**. Biotinylated NSCT-121 was generated using an EZ-Link NHS-PEG4-biotin kit and dialysed into PBS using a 7000Da MWCO Slide-A-Lyzer MINI Dialysis Unit (both Thermo Fisher Scientific). Human tenascin-C purified from a glioma cell line was purchased from Merck and fibrinogen purified from human plasma from Calbiochem.

**Table 2:** Summary of commercially available antibodies for detecting tenascin-C.

Antibody	Supplier	Species & isotype	Immunogen	Epitope	Reference
19C4MS	IBL	Mouse IgG1k	Recombinant FNIII A4-D	FNIIIC	(Hasegawa et al., 2004) (37)
4C8MS	IBL	Mouse IgG1K	Recombinant FNIII A4-D	FNIIIB	(Imanaka-Yoshida et al., 2002) (38)
4F10TT	IBL	Mouse IgG1	Purified glioma cell line tenascin-C	EGF-like repeats	(Tsunoda et al., 2003) (39)
4F8	Merck Millipore	Mouse IgG1k	Unknown	FNIII 4-5	–
9F8	Merck Millipore	Mouse IgG1K	Unknown	FNIII 1-3	(Ljubimov et al., 1998) (40)
NSCT-121	Nascient	Human IgG4K	Recombinant FBG	FBG	(Aungier et al., 2019) (36)
BC-24	Sigma- Aldrich	Mouse IgG1	Purified melanoma cell line tenascin-C	EGF-like repeats	(Siri et al., 1991) (41)
DB7	Merck Millipore	Mouse IgG2a	Purified foetal fibroblast tenascin-C	FBG	(Tiitta et al., 1992) (42)
EB2	Abcam	Mouse IgG1	Purified foetal fibroblast tenascin-C	FNIII 4-5	(Tervo et al., 1989) (43)
EPR4219	Abcam	Rabbit IgG	Recombinant FBG derived peptide	FBG	–
T2H5	Abcam	Mouse IgG1	Human mammary tumour homogenate	unknown	(Verstraeten et al., 1992) (44)

References (36, 38–45).

### 2.2 Patient samples

All human blood samples were provided by Axis-Shield Diagnostics Ltd (Dundee, Scotland) and were collected with informed consent in accordance with local ethical committee guidelines for the collection of research samples from human patients or donors. In total, 318 samples from separate individuals were used in this study including samples from 84 healthy donors, 150 RA patients, and 86 patients suffering from a range of diseases other than RA. Healthy donor samples of which 60% were women ranged from 19 to 74 years old with a mean age of 49 years. RA patients fulfilled the 1987 ACR/EULAR classification criteria for RA. RA patients ranged from 25 to 87 years old with a mean age of 58, of which 78% were women. Amongst RA donors, 103 were assessed for rheumatoid factor (RF) positivity of which 73 were RF positive and 30 were RF negative. Non-RA disease patients included 9 scleroderma patients, 9 osteoarthritis (OA) patients, 24 psoriatic arthritis patients, 14 ankylosing spondylitis patients, 7 systemic lupus erythematosus (SLE) patients, 4 polymyalgia rheumatic, 6 vasculitis, and 8 suffering from other miscellaneous rheumatic diseases. For diseased samples, of which 48% were women, ages ranged from 12 to 82 years old with a mean age of 55.

### 2.3 Tenascin-C sandwich ELISA

For all ELISAs, samples were run in duplicate with tenascin-C concentration calculated from a standard curve and fitted to standard samples using a quadratic polynomial regression, using Prism 8 statistical software (GraphPad Software, San Diego, USA). Data are presented as ng/ml total or splice-specific tenascin-C for all ELISAs.

Total tenascin-C was quantified in human serum and plasma samples using the following new sandwich ELISA. Mouse anti-tenascin-C monoclonal antibody, clone 9F8 (MAB1911; Merck, Darmstadt, Germany) was coated onto high-binding capacity 96 well plates (Biomat, Trento, Italy) at 2µg/ml in a carbonate buffer (15mM sodium carbonate, 35mM sodium bicarbonate, and pH 9.6) overnight at 2-8°C. The following day, the coating solution was decanted and plates were washed three times with phosphate-buffered saline 0.05% tween 20 (PBST) before blocking with 2% BSA in PBST for 1 hour at room temperature. Blocking solution was decanted and human serum or plasma samples were applied to the plate diluted at 1/200 in the sample buffer (1M NaCl, 4.9mM disodium phosphate, 0.5% Tween 20, 2% BSA, and pH 7.2) before incubation for 1 hour at room temperature. Following incubation, the sample solutions were decanted, and the plates were washed four times with PBST. Anti-tenascin-C monoclonal antibody, clone NSCT-121 conjugated to biotin and diluted at 5ng/ml in a conjugate buffer (PBS, 0.05% Tween 20, and 1% BSA) was used as a detection antibody. The detection antibody was applied to the plate for 1 hour at room temperature before decanting and washing four times with PBST. High sensitivity streptavidin-HRP (21130; Thermo Fisher Scientific) diluted at 0.2µg/ml in a conjugate buffer was then applied to the plate as a secondary detection reagent for 1 hour at room temperature. Following incubation, streptavidin-HRP was decanted and the plates were washed three times. Colourimetric detection was carried out using TMB solution (Moss Inc, Pasadena, USA) for 5 minutes before the colour change was quantified by measuring absorbance at 650nm using a Varioskan Flash Multimode Reader (Thermo Fisher Scientific). Pairwise screening of different combinations of anti-tenascin-C antibodies (**Table 2**) was performed using the same protocol. For competition assays, ELISA components were pre-incubated with detect, capture, or control antibodies for 30 min at 37°C before use.

To test the specificity of NSCT-121 Nunc, Immuno-Module F8 MaxiSorp plates (Thermo Fisher Scientific) were coated with 1μg/ml of purified human tenascin-C or fibrinogen overnight at 2-8°C. Plates were washed three times with phosphate-buffered saline 0.05% tween 20 (PBST) before blocking with 2% BSA in PBST for 1 hour at room temperature. Biotinylated detection antibody NSCT-121 was incubated at the concentrations stated for 1 hour at room temperature on an orbital plate shaker at 200 rpm. Secondary detection and colourimetric development were carried out with a streptavidin-HRP conjugate and TMB substrate solution.

To quantify splice-specific tenascin-C in human serum and plasma samples, the tenascin-C large FnIII-B and FnIII-C ELISA kits (IBL International, Hamburg, Germany) were used for the FnIII-B or FnIII-C splice variants, respectively. Both ELISAs were performed according to the manufacturer’s instructions with samples diluted at 1/400 for the FNIII-B assay and 1/10 for the FnIII-C assay.

### 2.4 Autoantibody sandwich ELISA

The detection of autoantibodies reactive to CCP2 or a previously identified citrullinated tenascin-C (cTNC) epitope (17) in human serum and plasma samples was carried out using an in-house ELISA. Biotinylated CCP2 (Axis-Shield Diagnostics Ltd, Dundee, UK), non-citrullinated TNC5 (rTNC5)(EHSIQFAEMKLRPSNFRNLEGRRKR), and citrullinated TNC5 (cTNC5) (EHSIQFAEMKL-cit-PSNF-cit-NLEG-cit-cit-KR) [both peptides produced by Pepceuticals Ltd (Leicester, UK)] peptides were coated onto Streptavidin Immobilizer 96 well plates (Nunc, Thermo Fisher Scientific) at 0.3µg/ml in a peptide buffer (PBST, 0.1% BSA) for 1 hour at room temperature. The peptide coating solution was then decanted, and the plates were washed three times before samples were applied in duplicate to the plate diluted at 1/100 in a sample buffer. Plates were incubated for 1 hour at room temperature after which the sample solutions were decanted and plates were washed three times. Bound autoantibody was then detected by the addition of anti-human IgG (γ-chain)-HRP (074–1002) or anti-human IgA (α-chain)-HRP (14–10–01) detection antibodies, both from Kirkegaard & Perry Laboratories (KPL; Gaithersburg, USA), and diluted to 0.1µg/ml in a conjugate buffer. Detection antibodies were applied to the plate for 1 hour at room temperature before decanting. Plates were washed three times before the addition of TMB HRP substrate solution (Moss Inc, Pasadena, USA) which was left to develop for 5 minutes. Colour change was then quantified by measuring absorbance at 650nm using a Varioskan Flash Multimode Reader (Thermo Fisher Scientific). Data for cTNC was presented as absorbance minus the absorbance value obtained for the rTNC coat. Data for CCP was presented as absorbance minus the absorbance value obtained for the no-peptide coat.

### 2.5 Tenascin-C immunoprecipitation from serum

Serum or plasma samples were diluted at 1/5 in PBS before pre-clearing by the addition of 2mg of magnetic M-270 streptavidin Dynabeads (Thermo Fisher Scientific) and then roll mixing for 1 hour. Following pre-clearing, the particles were brought to a magnet and the supernatant was transferred to another tube. Tenascin-C was then immunoprecipitated (IP) by the addition of 1mg of Dynabeads coupled to biotinylated tenascin-C specific NSCT-121 antibody (Nascient Ltd) before roller mixing at 4°C overnight. The following day, the Dynabeads were washed three times with an excess volume of PBS using a tube rotator and magnet. After the final wash, the Dynabeads were brought to the magnet and as much fluid was drained as possible before elution for western blotting or on-bead digestion for mass spectrometry analysis. For all samples, a matched control IP was also carried out using Dynabeads coated with an antibody to an irrelevant target to account for non-specific binding.

### 2.6 SDS-PAGE and western blotting

Samples were electrophoresed using a NuPAGE Novex PAGE system under denaturing conditions with buffers and equipment all provided by Thermo Fisher Scientific unless otherwise specified. Proteins were eluted from Dynabeads in 20µl 1x NuPAGE sample buffer supplemented with 1x NuPAGE reducing agent and denatured by incubation at 70°C for 10 minutes before cooling at room temperature. Samples were loaded on NuPAGE pre-cast 10% Bis-Tris protein gels in 1x NuPAGE MOPS SDS running buffer and electrophoresed at a constant 200V until the desired protein separation was achieved. Proteins were wet transferred at 30V for 90 minutes on ice onto Amersham Protran nitrocellulose membranes (GE Healthcare Life Sciences, Buckinghamshire, UK). Membranes were washed in PBST before blocking in Odyssey PBS Blocking Buffer (LI-COR Biotechnology, Cambridge, UK) for 1 hour at room temperature with agitation. Following blocking, the blocking buffer was discarded and 1µg/ml of B12 anti-tenascin-C primary antibody (Nascient Ltd.) was applied to the membrane in Odyssey PBS Blocking Buffer. Membranes were incubated with the primary antibody overnight at 4°C with agitation. The following day, the primary antibody solution was tipped off and the membranes were washed for 3 x 5 minutes in PBST (0.1%) at room temperature with agitation. Anti-mouse IgG-IRDye800 secondary antibody (926-32212; LI-COR Biotechnology) was then applied to the membrane at 0.3µg/ml in Odyssey PBS Blocking Buffer and incubated for 1 hour at room temperature with agitation. The secondary antibody solution was then discarded, and the membrane was washed for 3 x 5 minutes in PBST (0.1%) at room temperature with agitation. Blots were then visualised using an Odyssey imaging system (LI-COR Biotechnology) and captured scans were analysed using Image Studio Lite Ver 5.2 software (LI-COR Biotechnology).

### 2.7 Mass spectrometry

IP Dynabeads were resuspended in 70µl of MS buffer 1 (2M Urea, 50mM ammonium bicarbonate, and 1mM DTT), supplemented with 5µg/ml sequencing grade-modified trypsin (Promega, Madison, USA), and left to digest on a thermomixer at 29°C for 30 minutes at 1200 rpm. After digestion, a magnet was used to separate the beads, and the supernatant was taken off and transferred to another tube. The IP Dynabeads were then resuspended in 60µl of MS buffer 2 (2M urea, 50mM ammonium bicarbonate, and 5mM iodoacetamide), mixed, and separated using a magnet, and the supernatant was taken off. The second supernatant was pooled with the first supernatant and was then left to digest overnight and protected from light on a thermomixer at 37°C at 800 rpm. To stop the reaction, the next day, the sample was acidified by the addition of trifluoroacetic acid (TFA) for a final sample concentration of 0.5%.

Before mass spectrometric analysis, peptides from protein digests were first purified using C18 HyperSep SpinTip Microscale SPE Extraction Tips (Thermo Fisher Scientific). Briefly, columns were washed in acetonitrile buffer (80% Acetonitrile, 0.05% TFA) before equilibration in 0.05% TFA. Samples were passed through the column twice before washing with 0.05% TFA. Peptides were then eluted in acetonitrile buffer before sample drying in a Savant SPD 2010P1 speedvac (Thermo Fisher Scientific). Once dried, the sample was dissolved in formic acid and stored at -20°C until analysis by the Roslin Institute Proteomics and Metabolomics Facility by liquid chromatography-tandem mass spectrometry using an UltiMate HPLC system (Thermo Fisher Scientific) coupled to a micrOTOF-Q II ESI-Qq-TOF mass spectrometer (Bruker, Billerica, USA). Spectra were analysed using ProteinScape software (Bruker) in combination with the Mascot database search engine (Matrix Science, London, UK) for protein identification. To account for protein hits produced due to non-specific bead-binding, sample results were filtered to remove those with similar or higher scores in control non-specific IgG immunoprecipitations.

### 2.8 Statistical analysis

Statistical analysis was carried out using Prism 8 statistical software (GraphPad Software). For significance tests, P<0.05 was considered to be significant. Receiver operating characteristic (ROC) curves and likelihood ratios were used to assess test diagnostics. A p-value was calculated alongside ROC curve generation based on the area under the curve value, testing the null hypothesis that the diagnoses are made no better than random chance. Correlation between two variables was assessed using the non-parametric Spearman’s rank correlation test with a p-value also computed, testing the null hypothesis that the two variables were not correlated.

## 3 Results

### 3.1 The 9F8-NSCT sandwich ELISA detects full-length tenascin-C in human sera

A panel of commercially available antibodies detecting different sites within human tenascin-C was obtained (**Figure 1** and **Table 2**). Antibodies were screened and ranked for compatibility of detection of purified human tenascin-C in a 96-well plate sandwich ELISA format (**Table 3**). NSCT-121 was selected as the detection antibody and 9F8 as the capture antibody; this pairing was one of the more sensitive pairings screened with low background and effectively detects total tenascin-C levels. The epitopes for both antibodies in the FBG domain for NSCT-121 and the FNIII repeats 1-3 for 9F8 are constitutively expressed domains and are present in all forms of tenascin-C regardless of alternative splicing.

**Table 3:** Summary of antibody pair screening results.

Scale:	Not determined ND		NO Reactivity -	V. weak reactivity -/+	Weak reactivity +	Mild reactivity ++	Strong reactivity +++	V. strong reactivity ++++
Capture	Detection (HRP or biotin conjugated)							
	NSCT121 -HRP	NSCT121 -bio	9F8-HRP	4F10TT- HRP	T2H5. HRP	DB7-HRP	4C8MS- HRP	EPR4219 -HRP
NSCT121	-	-	++	++++	-	-	+/-	-
9F8	++	+++	-	++++	-	-	-	-
4F10TT	+	++	-	+	-	-	-	ND
T2H5	++	ND	+/-	ND	-	-	-	ND
DB7	+	ND	+/-	ND	-	-	+/-	ND
4C8MS	++	ND	+/-	++++ (kit)	-	-	-	ND
EPR4219	ND	-/+	+	ND	ND	ND	ND	ND
19C4MS	ND	+	ND	++++ (kit)	ND	ND	ND	ND

Antibody pairings were ranked on an arbitrary scale, indicated below, based on the specific absorbance signal they produced detecting purified tenascin-C. Some pairings were not tested and are marked as not determined. The results for the 4F10TT with 4C8MS and 19C4MS pairings reflect those obtained using commercial kits by IBL.

**Figure 2:**
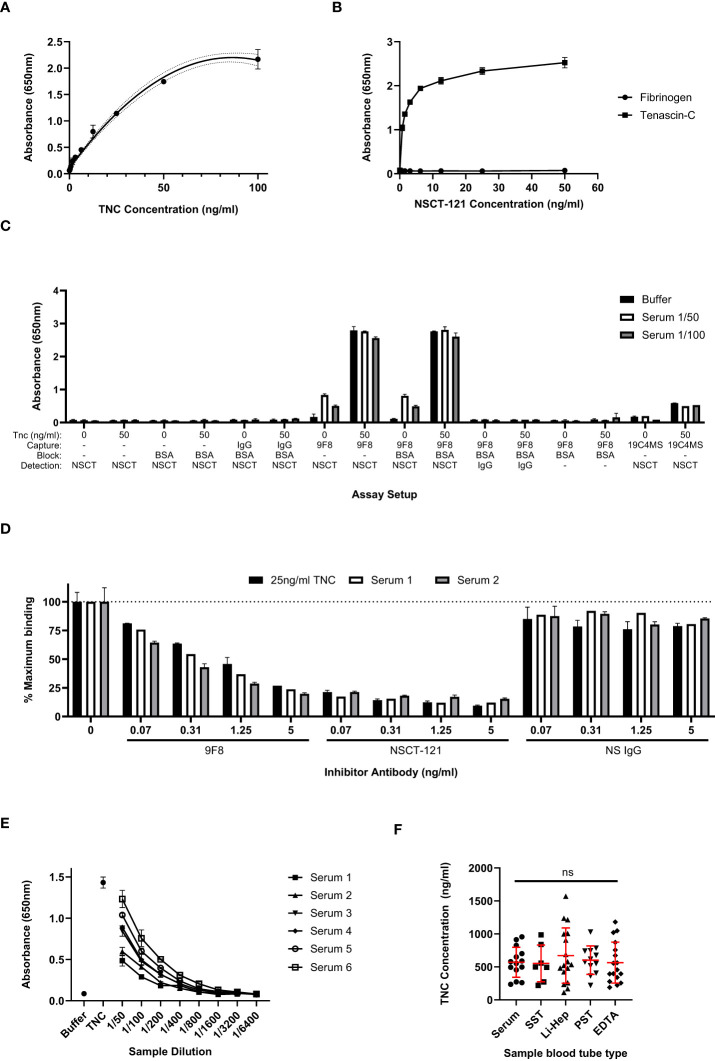
Total tenascin-C ELISA sensitivity and comparison with results obtained with splice-specific assays. **(A)** An extended standard curve for the total tenascin-C sandwich ELISA showing detection of purified tenascin-C from 0 to 100ng/ml. A linear dynamic range is observed between 0.78ng/ml to 50ng/ml. Data are presented as duplicate mean ± SD, dotted lines represent the 95% confidence limits. **(B)** Titration of biotinylated NSCT 121 antibody using 96 well plates coated with 1μg/ml human tenascin-C or fibrinogen. Data are shown as the mean +/-SD of duplicate experiments. **(C)** The swap-out experiment where different components of the 9F8-NSCT121 sandwich ELISA were removed or swapped to identify the source of the positive signal in the assay. Data are presented as duplicate mean +/-SD. **(D)** Antibody competitor assay whereby samples were either run with no competing antibody or in the presence of unlabelled competitor antibodies at the indicated concentrations. Only the tenascin-C specific 9F8 or NSCT121 antibodies abrogated recognition of tenascin-C in the serum samples or purified tenascin-C control. Data are calculated as a percentage inhibition with the no-inhibitor control set at 100%. Data are presented as the duplicate mean +/-SD. **(E)** Titration of six healthy control sera in the 9F8-NSCT121 sandwich ELISA. Buffer alone and 25ng/ml purified tenascin-C were used as positive and negative controls, respectively. Data are presented at the duplicate mean +/-SD. **(F)** Healthy donor samples were collected using different blood tube types, including serum (n=7), serum separator tube (SST; n=7), lithium heparin plasma (Li-Hep; n=17), plasma separator tube (PST; n=12), and EDTA plasma (n=17). Data are presented as points representing the mean of the individual’s sample’s duplicate, overlaid by the group mean +/- SD. Significance assessed by one-way ANOVA with Tukey’s multiple comparisons test, ns, not significant.

The sensitivity and dynamic range of the sandwich ELISA using these antibodies to detect total TNC was assessed with a titration of purified tenascin-C from 100ng/ml to 0ng/ml, as shown in **Figure 2A**. A lower limit of detection of 0.78ng/ml was determined, beyond which the tenascin-C dilutions were not reliably distinguishable from background levels. The linear dynamic range was determined to be between 0.78 and 50ng/ml and this was chosen as the concentration range for the assay standard curve going forward. From standard curves run across a number of plates, an average signal-to-noise ratio of 29:1 was calculated by dividing the absorbance signal of the highest standard by the background buffer-only readings. The specificity of the NSCT-121 antibody was verified, showing no detection of fibrinogen, a serum protein with homology to the C-terminal FBG domain of tenascin-C (**Figure 2B**), ruling out potential cross-reactivity amongst these proteins.

The assay was then evaluated for the detection of tenascin-C in serum samples. First, healthy control serum samples with or without added 50 ng/ml purified tenascin-C were tested in a ‘swap out’ experiment. This involved systematically removing or replacing components of the assay with non-specific reagents to ensure that the signal obtained from tenascin-C-spiked serum samples was specific and not the consequence of the non-specific binding of serum proteins. Signal above background was observed only when both tenascin-C-specific antibodies were used in the presence of tenascin-C-spiked serum and tenascin-C was prepared in a buffer or serum on its own (**Figure 2C**). This indicates that the assay specifically recognises the exogenous purified tenascin-C spiked into the samples, as well as endogenous tenascin-C present in the serum samples. No signal was detectable when using either NSCT-121 or 9F8 in combination with a non-specific IgG, and no reactivity with any other component of the assay, including plate or block, was detected. Additionally, the spike-recovery component of the assay also showed that analyte recovery was not affected by serum proteins. This was evident as compared to 50ng/ml purified tenascin-C in a buffer-alone recovery of tenascin-C; from the spiked 1/50 and 1/100, diluted serum was calculated as 98.5% ± 2.9% and 106.3% ± 4.2%, respectively.

To further validate the tenascin-C sandwich assay specificity, a competition assay was performed in which unlabelled 9F8, NSCT-121, or non-specific IgG were titrated into the assay samples in a concentration range from 5 to 0.07ng/ml. Both 9F8 and NSCT-121 dose-dependently reduced absorbance signal for both serum samples and buffer spiked with tenascin-C, whereas non-specific IgG had no effect on any of the samples tested. This suggests that the signal obtained in the tenascin-C sandwich assay was the result of the specific binding of 9F8 and NSCT-121 to tenascin-C (**Figure 2D**). The sample dilution factor for the assay was also assessed to ensure samples would fall within the standard curve range. A selection of sera from healthy controls containing varying basal levels of tenascin-C was serially diluted from 1/50 to 1/6400 and showed reasonable linearity of dilution. For this assay, 1/200 was selected as an appropriate sample dilution factor (**Figure 2E**).

Next, the blood sample tube type was assessed. The work so far utilised serum samples, which are produced by allowing the blood sample to clot before removing the clot and cells by centrifugation. However, plasma samples, to which anti-coagulation factors are added on sample collection to prevent clotting before centrifugation to remove cells, are also commonly used including different anti-coagulants such as lithium heparin and EDTA. Separator tubes, containing a gel that prevents the cell pellet from mixing with the supernatant after centrifugation, also exist for both plasma and serum samples. Screening of these sample types showed no significant difference in levels or distribution of tenascin-C indicating that sample collection does not impact assay performance (**Figure 2F**).

### 3.2 Elevated total serum tenascin-C is disease-agnostic, discriminating rheumatoid arthritis patients from healthy controls but with low specificity

**Figure 3:**
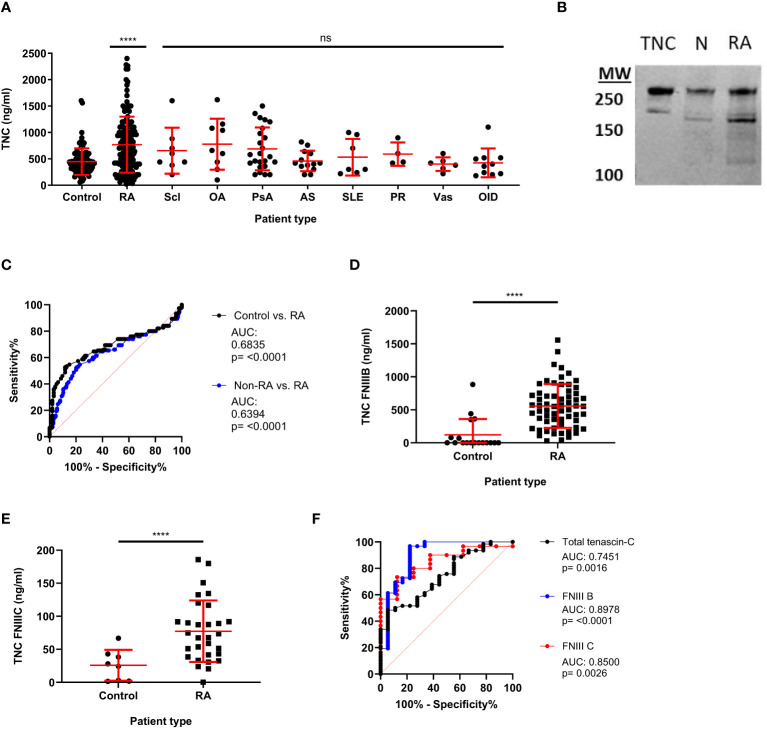
Total and splice-specific tenascin-C are significantly increased in the sera of rheumatoid arthritis patients compared to healthy controls. **(A)** Human blood samples were screened for tenascin-C using the total tenascin-C ELISA. Samples came from healthy donors (control) as well as, rheumatoid arthritis (RA), scleroderma (Scl), osteoarthritis (OA), psoriatic arthritis (PsA), ankylosing spondylitis (AS), systemic lupus erythematosus (SLE), polymyalgia rheumatica (PR), vasculitis (Vas), and other inflammatory diseases (OID) patients. n: Control = 84, RA = 150, Scl = 8, OA = 9, PsA = 24, AS = 13, SLE = 7, PR = 4, Vas = 6. Levels of total tenascin-C were higher in the RA group compared to the whole non-RA group (Mann-Whitney U test, p= 0.026) but levels were not significantly different between the RA group and any individual disease group including people with OA (Kruskal Wallis test, ns). **(B)** Western blot of immunoprecipitated tenascin-C from normal (N) and RA serum alongside 0.1µg of purified tenascin-C (TNC). Two major bands of corresponding heights to those seen in the purified tenascin-C are also seen in both the normal and RA IPs with the RA bands being more prominent. **(C)** Receiver operating characteristics (ROC) curve analysis for the diagnosis of RA patients (n=150) compared to healthy controls (n=93) or healthy controls and other rheumatic disease patients (non-RA; n=177) using the total tenascin-C ELISA. **(D, E)** Results for blood samples obtained from healthy donors (control) and RA patients for the tenascin-C FNIII-B (n: control = 18, RA = 62) **(D)** and FNIII-C (n: control = 8, RA = 30) **(E)** ELISAs. Data are presented as data points representing the mean of the individual sample’s technical duplicate overlaid by the group mean ± SD; Significance shown denotes the RA group **(A, D, E)** or whole non-RA group **(A)** compared to control determined by Kruskal-Wallis test **(A)**, Mann-Whitney U test **(D)**, and Student’s t-test **(E)**; **** = p<0.0001, ns = not significant. **(F)** Direct comparison between ROC curves obtained for the diagnosis of RA compared to healthy controls with the total tenascin-C, FNIII-B (n=control 18, RA = 62), or FNIII-C (n = 8, RA = 30) ELISAs. The dashed red line indicates the line of no discrimination.

Having established the total tenascin-C ELISA, screening was undertaken for a larger number of samples to assess levels of total tenascin-C in both healthy controls and patients suffering from a range of inflammatory or musculoskeletal diseases, including RA, scleroderma, OA, psoriatic arthritis, ankylosing spondylitis, SLE, polymyalgia rheumatic, and vasculitis. A moderate amount of basal tenascin-C was present in the serum of healthy controls, with an average of 447ng/ml detected. In line with its pro-inflammatory role, as well as previous reports on splice-specific tenascin-C, total tenascin-C was significantly increased in RA patients compared to healthy controls, with an average of 768ng/ml (p<0.0001). Amongst people with diseases other than RA, a number of patient groups showed a trend towards increased tenascin-C levels compared to healthy controls; none reached statistical significance although patient numbers were small for some diseases precluding any meaningful analysis between individual disease states. A comparison of the RA patients with the non-RA patients as a whole (n=81) showed the RA group had significantly higher total tenascin-C than other diseases (p=0.026); however, there was no significant difference between the RA group compared to any individual disease (**Figure 3A**).

The presence of tenascin-C in both healthy control and patient sera was further confirmed by immunoprecipitation followed by either mass spectrometry or western blotting for tenascin-C. Both methodologies confirmed the presence of tenascin-C with western blotting demonstrating that tenascin-C exists as two isoforms of approximately 160 and 280kDa in both healthy and RA patient samples (**Figure 3B**). Notably, the lower band in the standard, tenascin-C purified from human glioma cell lines, was larger than the lower band from either serum sample, suggesting that different splicing of tenascin-C occurs in these two sample types or different post-translational modification patterns, for example, glycosylation or proteolytic cleavage. Furthermore, there were additional lower molecular weight bands in the RA sample, suggesting that proteolytic cleavage of tenascin-C may occur in actively inflamed tissues with high enzymatic activity. The mass spectrometric analysis further identified that the ECM glycoprotein fibronectin co-immunoprecipitated with tenascin-C (**Table 4**).

**Table 4:** Proteins identified in mass spectrometric analysis of sera from healthy controls and RA sera tenascin-C IPs.

Protein ID	Protein name, species, and gene	Mascot Score
**Sample 1**	**Normal donor serum 1**	
F8W7G7	Fibronectin OS=Homo sapiens GN=FN1	1541
J3QSU6	Tenascin OS=Homo sapiens GN=TNC	595
**Sample 2**	**Normal donor serum 2**	
P02751	Fibronectin OS=Homo sapiens GN=FN1	2275
J3QSU6	Tenascin OS=Homo sapiens GN=TNC	190
**Sample 3**	**RA patient serum 1**	
J3QSU6	Tenascin OS=Homo sapiens GN=TNC	2073
P02751	Fibronectin OS=Homo sapiens GN=FN1	3045
**Sample 4**	**RA patient serum 2**	
J3QSU6	Tenascin OS=Homo sapiens GN=TNC	1361
F8W7G7	Fibronectin OS=Homo sapiens GN=FN1	307
**Sample 5**	**RA patient serum 3**	
P24821	Tenascin OS=Homo sapiens GN=TNC	2146
P02751	Fibronectin OS=Homo sapiens GN=FN1	798

Results were filtered by mascot score, a measure of confidence of identification, and by comparison to a non-specific IgG control IP.

ROC curve statistical analysis showed that total tenascin-C levels were able to discriminate RA patients from healthy controls with an AUC of 0.6835 (95% CI 0.6170 to 0.7501) (p<0.0001). When other diseases were included along with the healthy controls (non-RA group) in the analysis, the assay retained the ability to discriminate RA patients from non-RA patients and healthy controls; however, specificity decreased (AUC: 0.6394 (95% CI 0.5761 to 0.7027) (p<0.0001). Comparing only the non-RA disease group with RA, total tenascin-C was still able to discriminate the RA patients from other patients; however, with an AUC of 0.5887 (95% CI 0.5149 to 0.6625) (p=0.0262), specificity was even lower. To assess the specificity and sensitivity of total tenascin-C in discriminating people with RA from healthy controls, data extrapolated from the ROC curve show that using a cut-off based on the 99% or 97.5% percentile of the healthy controls, sensitivity was low (6.7 and 14% respectively), reaching 38% using a cut-off based on the 95% percentile of healthy controls (**Figure 3C** and **Table 5**).

**Table 5:** Specificity and sensitivity of the total tenascin-C assay in discriminating people with RA from healthy controls.

Cut-off % percentile	Cutoff [ng/ml]	Sensitivity	95% CI	Specificity	95% CI	Likelyhood Ratio
99	>1650	6.667	3.661% to 11.84%	100	96.03% to 100.0	
97.5	>1350	14	9.343% to 20.16%	97.85	92.49% to 99.62%	6.51
95	>850.0	38	30.62% to 52.00%	95.7	89.46% to 98.31%	8.835
92.5	>780.0	44	36.30% to 52.00%	91.4	83.93% to 95.58%	5.115
90	>740.0	46	38.22% to 53.98%	90.32	82.62% to 94.82%	4.753

Finally, a subset of healthy control and RA patients’ samples, covering a range of total tenascin-C concentrations, were selected for further screening for splice-specific tenascin-C. In line with previous publications, healthy controls were found to have mean sera concentrations of 121ng/ml and 25.7ng/ml of FnIII-B and FnIII-C, respectively, which, as for total tenascin-C levels, increased significantly to 557.8ng/ml and 77.3ng/ml in RA patients (**Figures 3D, E**). The diagnostic ability of the FnIII-B and FnIII-C splice variants was also assessed alongside the total tenascin-C ELISA in the subset of the samples screened with these assays. Both of these assays could discriminate the healthy donors from the RA patients with AUCs of 0.8978 (95% CI 0.7981 to 0.9976)(p<0.0001) for FNIII-B and 0.85 (95% CI 0.7205 to 0.9795)(p=0.0026) for FNIII-C. In this limited cohort, splice assays performed better than the total tenascin-C ELISA which had a lower AUC of 0.7451 (95% CI 0.6260 to 0.8642)(p=0.0016) (**Figure 3F**). Together, these data demonstrate that serum tenascin-C can discriminate patients from healthy controls and that disease specificity for both total and splice-specific tenascin-C was low, which is in line with literature showing this matrix molecule to be associated with a range of inflammatory diseases.

### 3.3 The distribution of total tenascin-C in human serum is distinct from specific splice variants and autoantibodies recognising citrullinated tenascin-C

**Figure 4:**
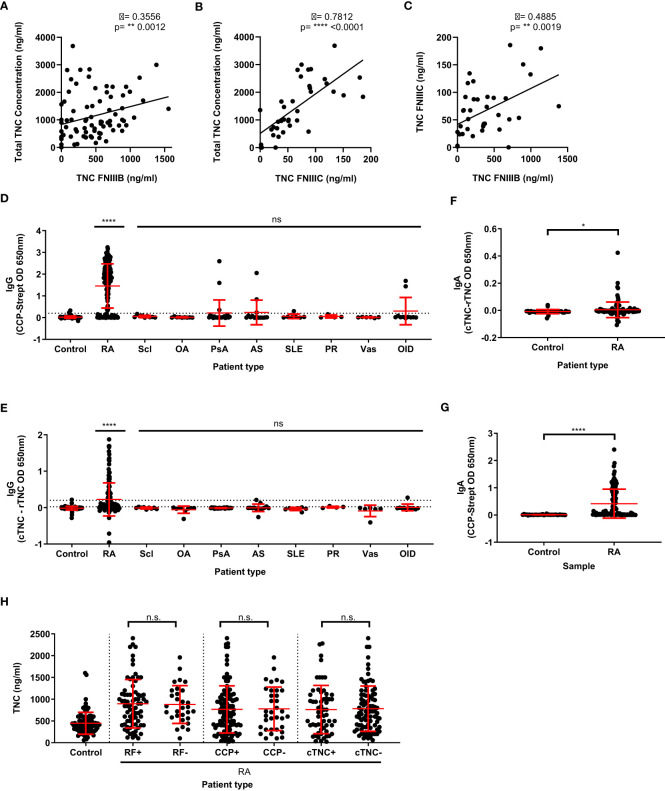
Total tenascin-C levels do not correlate with specific splice variants of tenascin-C, nor the presence of rheumatoid factor, anti-CCP, or anti-citrullinate tenascin-C autoantibodies. **(A, B)** Direct comparison of the total tenascin-C sandwich ELISA with the results obtained with the FNIII-B (n=80) **(A)** or the FNIII-C (n=38) **(B)** ELISAs. **(C)** Direct comparison between the FNIIIB and FNIIIC ELISA results (n=38). Data presented as data points representing the mean of the individual sample’s technical duplicate, significance determined by Spearman’s rank correlation analysis. **(D–G)** Human blood samples were screened for CCP-specific IgG **(D)** or IgA **(G)** autoantibodies and cTNC-specific IgG **(E)** or IgA **(F)** autoantibodies. Samples came from healthy donors (control) as well as rheumatoid arthritis (RA), scleroderma (Scl), osteoarthritis (OA), psoriatic arthritis (PsA), ankylosing spondylitis (AS), systemic lupus erythematosus (SLE), polymyalgia rheumatica (PR), vasculitis (Vas), and other inflammatory disease (OID) patients. For **(D)** and **(E)** n: Control = 82, RA = 150, Scl = 9, OA = 9, PsA = 24, AS = 14, SLE = 7, PR = 4, Vas = 6, OID = 11. For **(F)** and **(G)** n: Control = 33, RA = 112. **(H)** Results of the total tenascin-C ELISA for healthy donor controls or rheumatoid arthritis (RA) patients stratified by either rheumatoid factor (RF), cyclic citrullinated peptide (CCP), or cTNC5 peptide autoantibody positivity. n: control = 93, RF+ = 73, RF- = 30, CCP+ = 115, CCP- = 35, cTNC+ = 59, cTNC- = 88. Data presented as data points representing the mean of the individual sample’s technical duplicate overlaid by the group mean ± SD. Significance determined by Kruskal-Wallis test **(D, E, H)** or Mann-Whitney U test **(F, G)**, **** = p<0.0001, ** = p<0.01, * = p<0.05, ns, not significant.

Next, we assessed the relationship between total tenascin-C and splice-specific tenascin-C in human blood from a selection of healthy control, RA, and non-RA disease patient samples. Each was assayed for total tenascin-C using the sandwich ELISA and splice-specific tenascin-C, using two commonly employed ELISAs that specifically detect alternatively spliced domains FnIII-B and FnIII-C, respectively. The mean amounts of tenascin-C in patients assessed for total and both splice variants (± standard deviation) detected in each assay (1460.0 ± 968.9, 459.6 ± 362.2, and 66.4 ± 47.5 ng/ml for total, FnIII-B, and FnIII-C, respectively) differed greatly, indicating a substantial amount of tenascin-C in sera possessing neither splice variant. Correlation between the assays was also assessed and revealed that while total tenascin-C levels had a strong positive correlation (ρ=0.7812; p<0.0001) with FnIII-C containing tenascin-C, the correlation with FnIII-B containing tenascin-C levels (ρ=0.3556, p=0.0012) was weaker. Correlation between the different splice variants was likewise assessed with a similar level of disagreement found and only a moderate positive correlation (ρ=0.4885, p=0.0019) observed (**Figures 4A–C**). Together, these results demonstrate that splice-specific tenascin-C levels cannot be used as a proxy for total tenascin-C levels in human blood.

In addition to significant post-transcriptional modification, tenascin-C is also post-translationally modified by citrullination (18, 26, 46). Conversion of arginine residues to citrulline residues by this process is associated with a breach of tolerance in people with RA, leading to the generation of anti-citrullinated protein antibodies (ACPA), detection of which comprise one of the current gold standard diagnostic criteria for RA (47–49). As tenascin-C is known to be a target of ACPA, we investigated whether the levels of total tenascin-C correlated with antibody response. To do this, the levels of CCP IgG autoantibodies were first measured in our cohort by ELISA. Using a cut-off based on the 95% percentile of the healthy control, 116 RA patients were classified as positive (77.3%) for CCP autoantibodies compared to 13 patients or donors in all other conditions (15.5%). The level of anti-citrullinated tenascin-C cTNC5 autoantibodies was also measured in 78 RA patients (52%) and were classified as positive as opposed to 5 patients or donors in all other conditions (5.6%). All of these 78 cTNC5 positive RA patients were also CCP positive (**Figures 4D, E**).

IgG ACPA is not the only isotype reported with IgA autoantibodies also known to exist. As this isotype had not previously been investigated in the case of cTNC5 autoantibodies, we investigated IgA levels in a subset of patient samples. This was done by using the same ELISA assay adapted for IgA detection, with a cut-off based on the 95% percentile of the healthy control group for both CCP and cTNC sets to determine autoantibody positivity. For the RA sera, this resulted in 66/112 (58.9%) testing positive for CCP-specific IgA autoantibodies, which is in line with previous estimates of their prevalence in the literature (50). IgA autoantibodies to cTNC5 were also detected although in only 14/112 (12.5%) RA patients tested which, similarly to IgG, were all also CCP IgA positive (**Figures 4F, G**).

Using these data for ACPA autoantibodies, as well as data available for some patients on rheumatoid factor (RF) positivity, we determined if tenascin-C levels correlated with autoantibody positivity. Stratifying patients into either autoantibody-positive or negative groups revealed no significant differences in total tenascin-C levels between these groups (**Figure 4H**). Additional analysis further demonstrated no significant correlation between the absorbance levels in either the CCP or cTNC assays and total tenascin-C concentration (CCP ρ = -0.16, p=0.0527; cTNC ρ = -0.08, p=0.36). Together, these data highlight that none of the existing tools used to infer tenascin-C distribution in human clinical samples is a reliable proxy for directly assaying total tenascin-C itself.

## 4 Discussion

A wide range of serum tenascin-C concentrations in healthy and disease cohorts obtained using different assays have been reported in the literature. The majority of studies report an average tenascin-C concentration for healthy controls of 696ng/ml (9, 15, 51–57). This concentration is in reasonable agreement with tenascin-C concentrations we observed using the total tenascin-C sandwich ELISA developed in this study but is far greater than those observed with either of the two splice variant-specific ELISAs. Firstly, these data suggest that a notable amount of tenascin-C is expressed and transferred into the blood in normal healthy individuals, and secondly, that splice-specific detection ELISAs vastly underestimate circulating levels of this matrix molecule. The tissue or cellular source and functional significance of this serum tenascin-C remains to be elucidated.

In line with previous reports for splice variant-specific tenascin-C (16, 58), a total tenascin-C was elevated in the serum of the studied disease cohorts compared to healthy controls. This increase was only statistically significant for RA, which also had the highest levels of tenascin-C detected. These data likely reflect the smaller numbers of patients in each of the other disease groups studied. Accordingly, whilst a total tenascin-C could discriminate RA patients from healthy controls, the inclusion of the non-RA disease group significantly impacted assay specificity. This is in line with an increase in tenascin-C seen in many inflammatory diseases, suggesting it is more a marker of tissue inflammation than a specific disease such as RA. This is supported by previous studies of splice-specific tenascin-C which showed it correlated with the levels of the acute phase protein CRP, a general systemic marker of inflammatory responses (58). Low sensitivity at highly stringent specificity levels indicates that the clinical utility of this assay lies not in outright disease diagnosis but is better suited to identify people with an inflammatory disease in which tenascin-C contributes to disease progression and identify subsets of people that may be stratified for treatment with anti-tenascin-C antibodies. It is also important to consider the confounding factors that could impact tenascin-C levels when comparing disease groups. These include but are not limited to age, sex, ethnicity, disease duration or stage, treatment regime, and treatment response, which this study was not designed to assess. However, the development of this assay and validation in the patient samples used here paves the way for more extensive analysis of large, well-phenotyped patient cohorts that can be powered to interrogate the differences amongst these groups.

Splice-specific tenascin-C was also elevated in RA patient samples, and as in the healthy controls, total tenascin-C levels in RA patients were far greater than that found with the splice-specific ELISAs. However, the splice variant-specific assays performed better in ROC curve analysis than total tenascin-C in the limited number of patients and donors tested with all three assays. This suggests that the FNIII-B and FNIII-C splice variants may be more closely associated with the RA disease state. The biological role and receptor-binding partners for domains B and C are not well characterised. An overexpression of B promotes breast tumour cell proliferation and invasion (59, 60), whilst levels of FnIIIC+ variants positively correlate with disease duration and degree of joint erosion and negatively correlate with response to anti-TNF biologic therapy (16). However, the implications of their expression in inflammatory disease are not clear, warranting further functional investigation of these domains to complement existing biomarker studies.

Mass spectrometric analysis confirmed the presence of TNC in serum and further identified that it could be co-immunoprecipitated with the ECM glycoprotein fibronectin. These data are consistent with studies showing an interaction between these two matrix molecules in assays using recombinant or purified proteins and cellular matrices, for example, those derived from cultured fibroblasts (61–63), and demonstrate that circulating forms of these proteins may also interact in serum. This raises an interesting point around the detection of large, multi-modular, multi-ligand matrix proteins such as tenascin-C; not only is it important to think about the specific isoform/proteoform detected, but the issue of whether binding partners may preclude detection in biological samples is also key. The 9F8 antibody recognizes a site within FNIII repeats 1-3 of TNC. Purified plasma fibronectin can bind to recombinant proteins comprising different combinations of FNIII domains from TNC including 1-3 and 3-5 (63). Whilst neither the antibody epitope nor the fibronectin-binding site within FNIII repeats 1-3 have been further mapped, it is possible that 9F8 would not recognise TNC within a fibronectin-TNC complex. Whether FNIII (1–3)-mediated binding to fibronectin occurs *in situ* in serum is not yet known. Full-length TNC binds to the 70KDa C-terminal of fibronectin (63), a region that also binds to other fibronectin molecules, as well as to fibrin, heparin, collagen, and gelatin. Studies elucidating which of these proteins, all found at high levels in the circulation, form complexes and if and how these complexes change during disease onset and progression will be interesting to characterize.

Furthermore, matrix interactions are critically governed by proteolytic turnover. For example, full-length TNC does not interact with the cell-binding domain (CBD) of fibronectin, but smaller fragments, including FNIII repeats 1-3 do bind to this region (63). Therefore, analysis of the proteolytic state of circulating molecules will also be key going forward. This will be particularly important in the context of diseases such as RA where high tissue levels of matrix-degrading proteases such as MMPs may generate cleavage products that enter the circulation. The assay developed here will not detect fragments of TNC in which the N-terminal FNIII (1–3) domains have been liberated from the C-terminal FBG domain. At the last count, more than 50 ligands are known to bind TNC, and multiple potential proteolytic cleavage sites have been identified (3). Our assay provides an important step forward in quantitating total ‘free’, intact TNC, but there is still a great deal of work to be done before we have a complete picture of the distribution of this matrix molecule. Understanding more about the circulating forms of matrix proteins, for example, either within multiprotein complexes and/or as proteolytic cleavage products, will enable even more detailed interrogation of biologically relevant levels of these proteins. Indeed, studies that assess total collagen synthesis compared to collagen neoepitopes generated upon tissue destruction are proving useful in monitoring matrix homeostasis, repair, and turnover (2). One assay alone cannot capture this complexity: the development of additional assays that detect matrix complexes and matrix cleavage neoepitopes, alongside the detection of total unbound and uncleaved matrix proteoforms will very likely be the future state of the art in biomarker studies.

In samples where we directly compared total tenascin-C and isoform-targeted assays, the correlations between splice-specific variants and total tenascin-C were variable and, in most cases, weak. This suggests that splice-specific tenascin-C is not a good read out of total tenascin-C levels in serum and this should be taken into account when considering the results of the splice-specific assays. Additionally, stark differences were also observed between the two splice-specific assays and thus between the levels of the different splice variants. Only a modest correlation was observed, and the amount of the FNIII-C splice variant detected was much lower than the FNIII-B variant. While perhaps a reflection of differential assay performance, this could also suggest that the two exons are part of differential splicing modules resulting in different incorporation rates into tenascin-C and thus abundance. Alternatively, this could also suggest the differential ability of the spliceoforms to make it into circulation, perhaps due to the splicing conferring differential binding abilities or proteolytic processing.

Consideration should also be given to how accurately tenascin-C levels in the serum mirror the tenascin-C found at the pathological site as no current studies have characterised how tenascin-C ends up in circulation. This is additionally hampered by the fact that the majority of studies carried out on the tenascin-C found at the inflammatory site utilise non-quantitative methods of tenascin-C measurement such as immunohistochemistry or western blotting. RA is one of the few diseases where local amounts of tenascin-C have been quantified. Assessing tenascin-C concentrations in knee synovial fluid utilising the FNIII-C ELISA revealed that control joints contain very low amounts of tenascin-C with a median level of 7.7ng/ml, whereas joints from RA patients have much higher levels with a median of 166.8ng/ml (37, 45). While these studies did not investigate systemic tenascin-C, the data presented above for the FNIII-C ELISA found that control and RA patients had lower average systemic tenascin-C concentrations of 25.7 and 77.3ng/ml, respectively. While this might not be representative for other diseases or total tenascin-C, this does indicate that systemic tenascin-C does not necessarily completely match tissue levels of tenascin-C in disease.

Finally, screening of IgG ACPA in this study showed a prevalence of CCP and cTNC comparable to published data with similarly high specificity (17). Adaptation of the assay for IgA detection also demonstrated comparable specificity for RA patients, with 47.3% testing positive, which is in line with published studies (28, 50, 64). Analysis of tenascin-C levels in the context of RF factor positivity was also assessed in a subset of patients for whom these data were available. The tenascin-C sandwich ELISA used a mouse IgG capture designed to limit RF interference. Whilst this does not rule out potential RF cross-reactivity with other species IgG, the lack of correlation between RF positivity and tenascin-C concentration, the similar spread of the data between RF+ve and RF-ve RA patients, and no significant difference in the mean concentration between groups suggest that RF interference is not impacting the measurement of total TNC in this assay.

ACPA autoantibody positivity is associated with RA disease phenotypes. For example, CCP-negative patients have reduced synovial lymphocyte infiltration and more extensive fibrosis (65). As tenascin-C is itself a target of the auto-antibody response and has been found to modify both immune cell infiltration (28) and drive pro-fibrotic processes (66), we hypothesised that total tenascin-C levels may be associated with autoantibody subgroups. However, no relationship between any of the autoantibodies tested for total tenascin-C levels was found. While this may simply be evidence that tenascin-C is not mechanistically linked to autoantibody generation, this could also reflect the fact that antibodies arise well before disease onset, likely in extra-articular tissues such as the gut or lung, and serum tenascin-C levels may not accurately reflect this early pre-symptomatic process. Moreover, autoantibody formation is driven by genetic/environmental factors which may differ between patients who are positive for serum tenascin-C.

**Figure 5:**
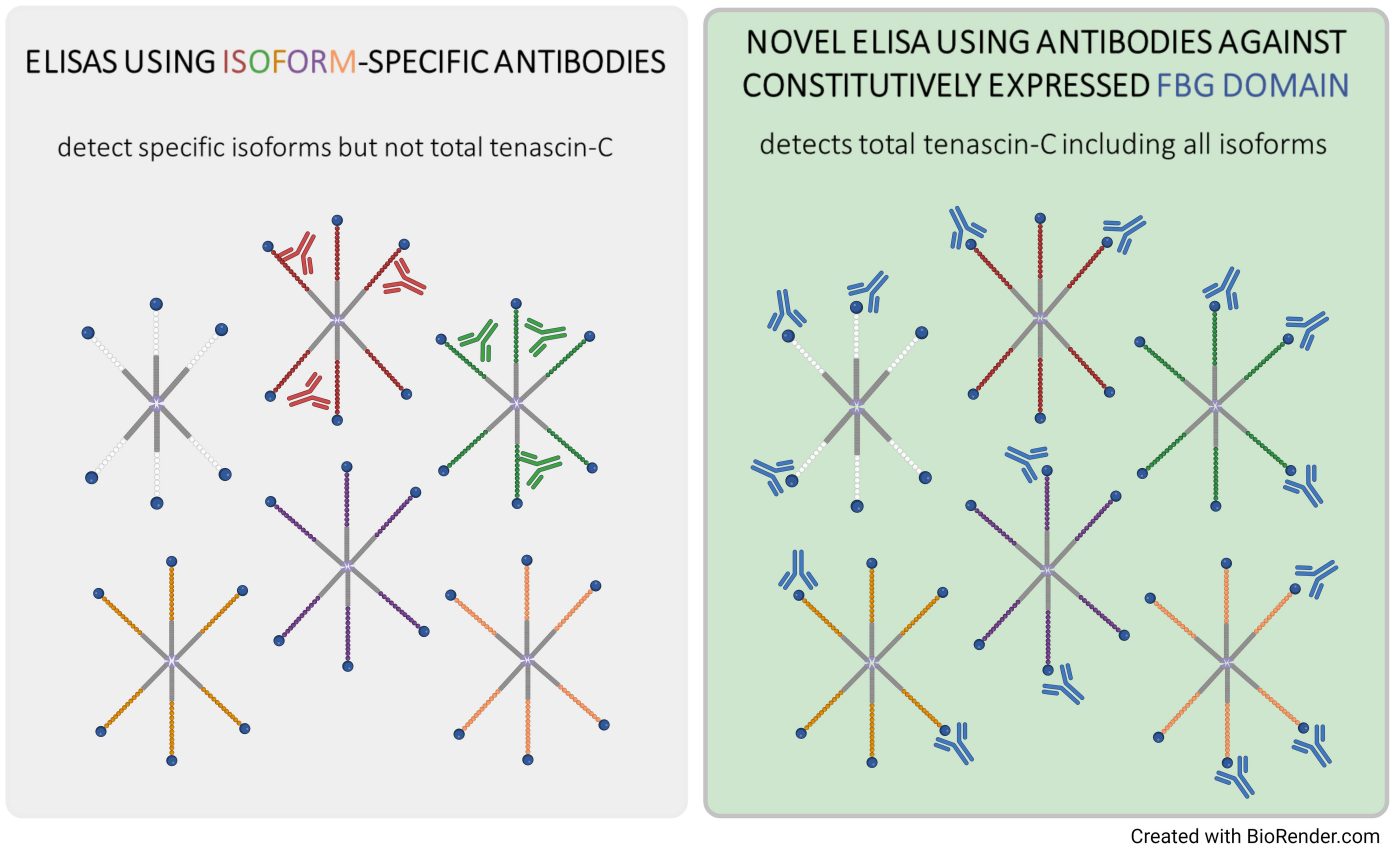
Summary cartoon comparing ELISA-based detection of tenascin-C in biological samples.

In summary, in this paper, we provide the first report of an immunoassay validated and optimised to assess total tenascin-C as a biomarker in clinical blood samples (**Figure 5**). Using this assay in a cohort of patients with inflammatory and musculoskeletal diseases, we observe differences in the distribution and levels of total tenascin-C compared to tenascin-C variants that raise intriguing questions about the role of distinct proteoforms in inflammatory disease. This assay now enables complementary analysis of total tenascin-C alongside splicing-targeted assays which pave the way for correlation of tenascin-C and specific isoforms with markers of inflammation, such as CRP or fibrinogen, and clinical data, for example, in RA including DAS28 score, tissue erosion, disease duration, treatment response, and cellular or molecular endotypes. Extending the application of this assay to other diseases, including different autoimmune conditions, fibrosis, and cancer, will also enable a better understanding of the complex patterns of expression of tenascin-C and provide new insight into the inflammatory status across a wider pathology. The development of this methodology also highlights that as interest in multimodular matrix molecules in general and tenascin-C in particular in inflammatory disease diagnosis and treatment is exponentially expanding, careful assay selection is required to accurately quantify their tissue distribution.

## Data availability statement

The original contributions presented in the study are included in the article/supplementary material, further inquiries can be directed to the corresponding author/s.

## Ethics statement

Ethical approval was obtained by Axis Shield Ltd. The studies were conducted in accordance with the local legislation and institutional requirements. The human samples used in this study were acquired from gifted from another research group. Written informed consent for participation was not required from the participants or the participants’ legal guardians/next of kin in accordance with the national legislation and institutional requirements.

## Author contributions

JO: Data curation, Writing – original draft, Writing – review & editing, Formal analysis, Investigation, Methodology. ML: Formal analysis, Investigation, Methodology, Supervision, Writing – review & editing. AS: Formal analysis, Methodology, Writing – review & editing. DK: Formal analysis, Methodology, Writing – review & editing, Investigation. JB: Formal Analysis, Writing – review & editing, Supervision. DP: Formal analysis, Supervision, Writing – review & editing, Methodology. GM: Formal analysis, Supervision, Writing – review & editing. CF: Formal analysis, Supervision, Writing – review & editing, Funding acquisition, Project administration, Resources. KM: Conceptualization, Data curation, Funding acquisition, Project administration, Resources, Supervision, Writing – original draft, Writing – review & editing.

## References

[1] Buckley CD Ospelt C Gay S Midwood KS. Location, location, location: how the tissue microenvironment affects inflammation in RA. Nat Rev Rheumatol (2021) 17(4):195–212. doi: 10.1038/s41584-020-00570-2 33526927

[2] Alexdottir MS Bourgonje AR Karsdal MA Pehrsson M Loveikyte R van Dullemen HM. Serological biomarkers of intestinal collagen turnover identify early response to infliximab therapy in patients with crohn's disease. Front Med (Lausanne). (2022) 9:933872. doi: 10.3389/fmed.2022.933872 35903311 PMC9315105

[3] Midwood KS Chiquet M Tucker RP Orend G. Tenascin-C at a glance. J Cell Sci (2016) 129(23):4321–7. doi: 10.1242/jcs.190546 27875272

[4] Spenle C Saupe F Midwood K Burckel H Noel G Orend G. Tenascin-C: Exploitation and collateral damage in cancer management. Cell adhesion migration (2015) 9(1-2):141–53. doi: 10.1080/19336918.2014.1000074 PMC4422814 25569113

[5] Udalova IA Ruhmann M Thomson SJ Midwood KS. Expression and immune function of tenascin-C. Crit Rev Immunol (2011) 31(2):115–45. doi: 10.1615/CritRevImmunol.v31.i2.30 21542790

[6] Ishizaki J Takemori A Suemori K Matsumoto T Akita Y Sada KE. Targeted proteomics reveals promising biomarkers of disease activity and organ involvement in antineutrophil cytoplasmic antibody-associated vasculitis. Arthritis Res Ther (2017) 19(1):218. doi: 10.1186/s13075-017-1429-3 28962592 PMC5622475

[7] Yasuda M Harada N Harada S Ishimori A Katsura Y Itoigawa Y. Characterization of tenascin-C as a novel biomarker for asthma: utility of tenascin-C in combination with periostin or immunoglobulin E. Allergy Asthma Clin Immunol (2018) 14:72. doi: 10.1186/s13223-018-0300-7 30473714 PMC6241046

[8] Gupta L Bhattacharya S Aggarwal A. Tenascin-C, a biomarker of disease activity in early ankylosing spondylitis. Clin Rheumatol (2018) 37(5):1401–5. doi: 10.1007/s10067-017-3938-5 29313272

[9] Riedl S Tandara A Reinshagen M Hinz U Faissner A Bodenmuller H. Serum tenascin-C is an indicator of inflammatory bowel disease activity. Int J colorectal disease. (2001) 16(5):285–91. doi: 10.1007/s003840100312 11686525

[10] Muller S Neureiter D Stolte M Verbeke C Heuschmann P Kirchner T. Tenascin: a sensitive and specific diagnostic marker of minimal collagenous colitis. Virchows Arch (2001) 438(5):435–41. doi: 10.1007/s004280000375 11407470

[11] Muller-Felber W Toepfer M Muller T Muller-Hocker J Fischer P Lochmuller H. Tenascin is a useful marker in the diagnosis of inflammatory myopathies. Eur J Med Res (1998) 3(6):281–7. 9620889

[12] Shukla A Gaur P Aggarwal A. Tenascin-C levels, A toll-like receptor 4 ligand, in enthesitis-related arthritis category of juvenile idiopathic arthritis: A cross-sectional and longitudinal study. J Rheumatol (2015) 42(5):891–6. doi: 10.3899/jrheum.141365 25774061

[13] Morimoto S Imanaka-Yoshida K Hiramitsu S Kato S Ohtsuki M Uemura A. Diagnostic utility of tenascin-C for evaluation of the activity of human acute myocarditis. J Pathol (2005) 205(4):460–7. doi: 10.1002/path.1730 15685595

[14] Tsukada B Terasaki F Shimomura H Otsuka K Otsuka K Katashima T. High prevalence of chronic myocarditis in dilated cardiomyopathy referred for left ventriculoplasty: expression of tenascin C as a possible marker for inflammation. Hum Pathol (2009) 40(7):1015–22. doi: 10.1016/j.humpath.2008.12.017 19297005

[15] Latijnhouwers MA Bergers M Kuijpers AL van der Vleuten CJ Dijkman H van de Kerkhof PC. Tenascin-C is not a useful marker for disease activity in psoriasis. Acta Derm Venereol. (1998) 78(5):331–4. doi: 10.1080/000155598442980 9779247

[16] Page TH Charles PJ Piccinini AM Nicolaidou V Taylor PC Midwood KS. Raised circulating tenascin-C in rheumatoid arthritis. Arthritis Res Ther (2012) 14(6):R260. doi: 10.1186/ar4105 23193984 PMC3674624

[17] Schwenzer A Jiang X Mikuls TR Payne JB Sayles HR Quirke AM. Identification of an immunodominant peptide from citrullinated tenascin-C as a major target for autoantibodies in rheumatoid arthritis. Ann Rheum Dis (2015). doi: 10.1136/annrheumdis-2015-208495 PMC5036245 26659718

[18] Raza K Schwenzer A Juarez M Venables P Filer A Buckley CD. Detection of antibodies to citrullinated tenascin-C in patients with early synovitis is associated with the development of rheumatoid arthritis. RMD Open (2016) 2(2):e000318. doi: 10.1136/rmdopen-2016-000318 27933208 PMC5133409

[19] Schwenzer A Quirke AM Marzeda AM Wong A Montgomery AB Sayles HR. Association of distinct fine specificities of anti-citrullinated peptide antibodies with elevated immune responses to prevotella intermedia in a subgroup of patients with rheumatoid arthritis and periodontitis. Arthritis Rheumatol (2017) 69(12):2303–13. doi: 10.1002/art.40227 PMC5711558 29084415

[20] Brissett M Veraldi KL Pilewski JM Medsger TA Jr. Feghali-Bostwick CA. Localized expression of tenascin in systemic sclerosis-associated pulmonary fibrosis and its regulation by insulin-like growth factor binding protein 3. Arthritis Rheumatol (2012) 64(1):272–80. doi: 10.1002/art.30647 PMC3241902 21898349

[21] Zavada J Uher M Svobodova R Olejarova M Husakova M Ciferska H. Serum tenascin-C discriminates patients with active SLE from inactive patients and healthy controls and predicts the need to escalate immunosuppressive therapy: a cohort study. Arthritis Res Ther (2015) 17:341. doi: 10.1186/s13075-015-0862-4 26608564 PMC4660660

[22] Latijnhouwers MA Pfundt R de Jongh GJ Schalkwijk J. Tenascin-C expression in human epidermal keratinocytes is regulated by inflammatory cytokines and a stress response pathway. Matrix Biol (1998) 17(4):305–16. doi: 10.1016/S0945-053X(98)90083-X 9749946

[23] Masaki T Yorioka N Taniguchi Y Oda H Yamakido M. Tenascin expression may reflect the activity and chronicity of human IgA nephropathy. Clin Nephrol. (1998) 50(4):205–13. 9799064

[24] Okuma Y Suda K Nakaoka H Katsube Y Mitani Y Yoshikane Y. Serum tenascin-C as a novel predictor for risk of coronary artery lesion and resistance to intravenous immunoglobulin in kawasaki disease- A multicenter retrospective study. Circ J (2016) 80(11):2376–81. doi: 10.1253/circj.CJ-16-0563 27746411

[25] Marzeda AM Midwood KS. Internal affairs: tenascin-C as a clinically relevant, endogenous driver of innate immunity. J Histochem Cytochem (2018) 66(4):289–304. doi: 10.1369/0022155418757443 29385356 PMC5958381

[26] Song J Schwenzer A Wong A Turcinov S Rims C Martinez LR. Shared recognition of citrullinated tenascin-C peptides by T and B cells in rheumatoid arthritis. JCI Insight (2021) 6(5). doi: 10.1172/jci.insight.145217 PMC8021118 33507879

[27] Giblin SP Midwood KS. Tenascin-C: Form versus function. Cell adhesion migration (2015) 9(1-2):48–82. doi: 10.4161/19336918.2014.987587 25482829 PMC4422809

[28] Giblin SP Schwenzer A Midwood KS. Alternative splicing controls cell lineage-specific responses to endogenous innate immune triggers within the extracellular matrix. Matrix Biol (2020) 93:95–114. doi: 10.1016/j.matbio.2020.06.003 32599145

[29] Tanaka R Seki Y Saito Y Kamiya S Fujita M Okutsu H. Tenascin-C-derived peptide TNIIIA2 highly enhances cell survival and platelet-derived growth factor (PDGF)-dependent cell proliferation through potentiated and sustained activation of integrin alpha5beta1. J Biol Chem (2014) 289(25):17699–708. doi: 10.1074/jbc.M113.546622 PMC4067204 24808173

[30] Meiners S Nur-e-Kamal MS Mercado ML. Identification of a neurite outgrowth-promoting motif within the alternatively spliced region of human tenascin-C. J Neurosci (2001) 21(18):7215–25. doi: 10.1523/JNEUROSCI.21-18-07215.2001 PMC6762977 11549732

[31] Mercado ML Nur-e-Kamal A Liu HY Gross SR Movahed R Meiners S. Neurite outgrowth by the alternatively spliced region of human tenascin-C is mediated by neuronal alpha7beta1 integrin. J Neurosci (2004) 24(1):238–47. doi: 10.1523/JNEUROSCI.4519-03.2004 PMC6729556 14715956

[32] Fouda GG Jaeger FH Amos JD Ho C Kunz EL Anasti K. Tenascin-C is an innate broad-spectrum, HIV-1-neutralizing protein in breast milk. Proc Natl Acad Sci U S A. (2013) 110(45):18220–5. doi: 10.1073/pnas.1307336110 PMC3831436 24145401

[33] Mangan RJ Stamper L Ohashi T Eudailey JA Go EP Jaeger FH. Determinants of Tenascin-C and HIV-1 envelope binding and neutralization. Mucosal Immunol (2019) 12(4):1004–12. doi: 10.1038/s41385-019-0164-2 PMC6599478 30976088

[34] Mansour RG Stamper L Jaeger F McGuire E Fouda G Amos J. The presence and anti-HIV-1 function of tenascin C in breast milk and genital fluids. PloS One (2016) 11(5):e0155261. doi: 10.1371/journal.pone.0155261 27182834 PMC4868279

[35] Frey K Fiechter M Schwager K Belloni B Barysch MJ Neri D. Different patterns of fibronectin and tenascin-C splice variants expression in primary and metastatic melanoma lesions. Exp Dermatol (2011) 20(8):685–8. doi: 10.1111/j.1600-0625.2011.01314.x 21649738

[36] Aungier SR Cartwright AJ Schwenzer A Marshall JL Dyson MR Slavny P. Targeting early changes in the synovial microenvironment: a new class of immunomodulatory therapy? Ann Rheum Dis (2019) 78(2):186–91. doi: 10.1136/annrheumdis-2018-214294 PMC6352652 30552174

[37] Hasegawa M Hirata H Sudo A Kato K Kawase D Kinoshita N. Tenascin-C concentration in synovial fluid correlates with radiographic progression of knee osteoarthritis. J Rheumatol (2004) 31(10):2021–6. 15468369

[38] Imanaka-Yoshida K Hiroe M Yasutomi Y Toyozaki T Tsuchiya T Noda N. Tenascin-C is a useful marker for disease activity in myocarditis. J Pathol (2002) 197(3):388–94. doi: 10.1002/path.1131 12115886

[39] Tsunoda T Inada H Kalembeyi I Imanaka-Yoshida K Sakakibara M Okada R. Involvement of large tenascin-C splice variants in breast cancer progression. Am J Pathol (2003) 162(6):1857–67. doi: 10.1016/S0002-9440(10)64320-9 PMC1868127 12759243

[40] Ljubimov AV Saghizadeh M Spirin KS Khin HL Lewin SL Zardi L. Expression of tenascin-C splice variants in normal and bullous keratopathy human corneas. Invest Ophthalmol Vis Sci (1998) 39(7):1135–42. 9620072

[41] Siri A Carnemolla B Saginati M Leprini A Casari G Baralle F. Human tenascin: primary structure, pre-mRNA splicing patterns and localization of the epitopes recognized by two monoclonal antibodies. Nucleic Acids Res (1991) 19(3):525–31. doi: 10.1093/nar/19.3.525 PMC333643 1707164

[42] Tiitta O Wahlstrom T Paavonen J Linnala A Sharma S Gould VE. Enhanced tenascin expression in cervical and vulvar koilocytotic lesions. Am J Pathol (1992) 141(4):907–13. PMC1886622 1384340

[43] Tervo K Tervo T van Setten GB Tarkkanen A Virtanen I. Demonstration of tenascin-like immunoreactivity in rabbit corneal wounds. Acta Ophthalmol (Copenh). (1989) 67(3):347–50. doi: 10.1111/j.1755-3768.1989.tb01886.x 2475011

[44] Verstraeten AA Mackie EJ Hageman PC Hilgers J Schol DJ De Jongh GJ. Tenascin expression in basal cell carcinoma. Br J Dermatol (1992) 127(6):571–4. doi: 10.1111/j.1365-2133.1992.tb14867.x 1282358

[45] Hasegawa M Nakoshi Y Muraki M Sudo A Kinoshita N Yoshida T. Expression of large tenascin-C splice variants in synovial fluid of patients with rheumatoid arthritis. J orthopaedic Res (2007) 25(5):563–8. doi: 10.1002/jor.20366 17262825

[46] Schwenzer A Jiang X Mikuls TR Payne JB Sayles HR Quirke AM. Identification of an immunodominant peptide from citrullinated tenascin-C as a major target for autoantibodies in rheumatoid arthritis. Ann Rheum Dis (2016) 75(10):1876–83. doi: 10.1136/annrheumdis-2015-208495 PMC5036245 26659718

[47] Aletaha D Neogi T Silman AJ Funovits J Felson DT Bingham CO 3rd. 2010 Rheumatoid arthritis classification criteria: an American College of Rheumatology/European League Against Rheumatism collaborative initiative . Arthritis Rheum (2010) 62(9):2569–81. doi: 10.1002/art.27584 20872595

[48] Demoruelle MK Deane K. Antibodies to citrullinated protein antigens (ACPAs): clinical and pathophysiologic significance. Curr Rheumatol Rep (2011) 13(5):421–30. doi: 10.1007/s11926-011-0193-7 PMC4095867 21713412

[49] Song YW Kang EH. Autoantibodies in rheumatoid arthritis: rheumatoid factors and anticitrullinated protein antibodies. QJM monthly J Assoc Physicians. (2010) 103(3):139–46. doi: 10.1093/qjmed/hcp165 PMC2825384 19926660

[50] Lakos G Soos L Fekete A Szabo Z Zeher M Horvath IF. Anti-cyclic citrullinated peptide antibody isotypes in rheumatoid arthritis: association with disease duration, rheumatoid factor production and the presence of shared epitope. Clin Exp Rheumatol (2008) 26(2):253–60. 18565246

[51] Yamauchi M Mizuhara Y Maezawa Y Toda G. Serum tenascin levels in chronic liver disease. Liver (1994) 14(3):148–53. doi: 10.1111/j.1600-0676.1994.tb00064.x 7521506

[52] Ylatupa S Mertaniemi P Haglund C Partanen P. Enzyme immunoassay for quantification of tenascin in biologic samples. Clin Biochem (1995) 28(3):263–8. doi: 10.1016/0009-9120(94)00079-B 7554244

[53] Kaarteenaho-Wiik R Mertaniemi P Sajanti E Soini Y Paakko P. Tenascin is increased in epithelial lining fluid in fibrotic lung disorders. Lung (1998) 176(6):371–80. doi: 10.1007/PL00007619 9780295

[54] Riedl S Bodenmuller H Hinz U Holle R Moller P Schlag P. Significance of tenascin serum level as tumor marker in primary colorectal carcinoma. Int J Cancer. (1995) 64(1):65–9. doi: 10.1002/ijc.2910640113 7545144

[55] Kimura S Ishida S Matunaga K Washizu K Hiraiwa H Takeuchi K. Determination of tenascin-C in human serum by the use of a new enzyme immunoassay. Biomed Res (1993) 14(3):203–8. doi: 10.2220/biomedres.14.203

[56] Washizu K Kimura S Hiraiwa H Matsunaga K Kuwabara M Ariyoshi Y. Development and application of an enzyme immunoassay for tenascin. Clin Chim Acta (1993) 219(1-2):15–22. doi: 10.1016/0009-8981(93)90193-8 7508343

[57] Yoshida J Wakabayashi T Okamoto S Kimura S Washizu K Kiyosawa K. Tenascin in cerebrospinal fluid is a useful biomarker for the diagnosis of brain tumour. J Neurol Neurosurg Psychiatry (1994) 57(10):1212–5. doi: 10.1136/jnnp.57.10.1212 PMC485489 7523604

[58] Schenk S Muser J Vollmer G Chiquet-Ehrismann R. Tenascin-C in serum: a questionable tumor marker. Int J Cancer. (1995) 61(4):443–9. doi: 10.1002/ijc.2910610402 7538974

[59] Guttery DS Hancox RA Mulligan KT Hughes S Lambe SM Pringle JH. Association of invasion-promoting tenascin-C additional domains with breast cancers in young women. Breast Cancer Res (2010) 12(4):R57. doi: 10.1186/bcr2618 20678196 PMC2949648

[60] Hancox RA Allen MD Holliday DL Edwards DR Pennington CJ Guttery DS. Tumour-associated tenascin-C isoforms promote breast cancer cell invasion and growth by matrix metalloproteinase-dependent and independent mechanisms. Breast Cancer Res (2009) 11(2):R24. doi: 10.1186/bcr2251 19405959 PMC2688953

[61] Chung CY Zardi L Erickson HP. Binding of tenascin-C to soluble fibronectin and matrix fibrils. J Biol Chem (1995) 270(48):29012–7. doi: 10.1074/jbc.270.48.29012 7499434

[62] To WS Midwood KS. Cryptic domains of tenascin-C differentially control fibronectin fibrillogenesis. Matrix Biol (2010) 29(7):573–85. doi: 10.1016/j.matbio.2010.08.003 20708078

[63] To WS Midwood KS. Identification of novel and distinct binding sites within tenascin-C for soluble and fibrillar fibronectin. J Biol Chem (2011) 286(17):14881–91. doi: 10.1074/jbc.M110.189019 PMC3083171 21324901

[64] Verpoort KN Jol-van der Zijde CM Papendrecht-van der Voort EA Ioan-Facsinay A Drijfhout JW van Tol MJ. Isotype distribution of anti-cyclic citrullinated peptide antibodies in undifferentiated arthritis and rheumatoid arthritis reflects an ongoing immune response. Arthritis Rheumatol (2006) 54(12):3799–808. doi: 10.1002/art.22279 17133560

[65] van Oosterhout M Bajema I Levarht EW Toes RE Huizinga TW van Laar JM. Differences in synovial tissue infiltrates between anti-cyclic citrullinated peptide-positive rheumatoid arthritis and anti-cyclic citrullinated peptide-negative rheumatoid arthritis. Arthritis Rheumatol (2008) 58(1):53–60. doi: 10.1002/art.23148 18163491

[66] Bhattacharyya S Wang W Morales-Nebreda L Feng G Wu M Zhou X. Tenascin-C drives persistence of organ fibrosis. Nat Commun (2016) 7:11703. doi: 10.1038/ncomms11703 27256716 PMC4895803

